# Outbreak of foodborne disease in a boarding school, Negeri Sembilan state, Malaysia, 2021

**DOI:** 10.5365/wpsar.2023.1.4.3.1043

**Published:** 2019-09-30

**Authors:** Nur Nadiatul Asyikin Bujang, Mohd Shahrol Abd Wahil, Siti Aishah Abas, Khairul Hafidz Alkhair Khairul Amin, Nadiatul Ima Zulkifli, Sharina Mohd Shah, Nurul Fazilah Aziz, Syuaib Aiman Amir Kamarudin, Veshny Ganesan, Nur Azieanie Zainuddin, Muhamad Hazizi Muhamad Hasani, Noor Khalili Mohd Ali, Mohamad Paid Yusof

**Affiliations:** a Seremban District Health Office, Ministry of Health, Seremban, Negeri Sembilan, Malaysia.; b Department of Social and Preventive Medicine, Faculty of Medicine, Universiti Malaya, Kuala Lumpur, Malaysia.; c Disease Control Division, Ministry of Health, Putrajaya, Malaysia.; d Department of Public Health and Population Medicine, Faculty of Medicine, Universiti Teknologi MARA, Shah Alam, Selangor, Malaysia.; e Department of Community Health, Faculty of Medicine and Health Sciences, Universiti Putra Malaysia, Serdang, Selangor, Malaysia.

## Abstract

**Objective:**

Foodborne disease is a significant global public health concern, with *Bacillus cereus* being a frequent cause of outbreaks. However, due to the relatively mild symptoms caused by infection with *B. cereus*, the shorter duration of illness and the challenges of testing for it in both stool and food samples, outbreaks are often underreported. This report describes the epidemiology of cases of foodborne illness, the causative agent and risk factors associated with an outbreak in a boarding school in Seremban district, Negeri Sembilan state, Malaysia, that occurred in November 2021.

**Methods:**

Epidemiological, environmental and laboratory investigations were performed. A case was defined as any person with abdominal pain, vomiting or diarrhoea that occurred after consuming food served by the canteen at the school. The data were analysed using Microsoft Excel and the Statistical Package for the Social Sciences (SPSS).

**Results:**

A total of 152 cases were identified among the 597 students, giving an attack rate of 25.5%. All cases were females aged 13–17 years. They presented with abdominal pain (100%), nausea (97.4%, 148), vomiting (78.3%, 119) or diarrhoea (61.8%, 94), or a combination of these. The mode of transmission of the outbreak was a continual common source. The foods associated with becoming a case were beef rendang (a dry curry) (odds ratio [OR]: 20.54, 95% CI: 4.89–86.30), rice (OR: 19.62, 95% CI: 2.62–147.01), rice cubes (OR: 18.17, 95% CI: 4.31–76.55) and vermicelli (OR: 17.02, 95% CI: 4.03–71.86). Cross-contamination and inadequate thawing and storage temperatures contributed to the outbreak.

**Discussion:**

This outbreak of foodborne illness at a boarding school was likely caused by *B. cereus*. The findings highlight the importance of proper food preparation, temperature monitoring, hygiene practices among food handlers and compliance with food safety guidelines.

Foodborne diseases continue to be a public health concern globally, contributing to morbidity and mortality, and associated with substantial economic costs. (*1*) During the past decade, the national annual incidence of foodborne diseases in Malaysia has been decreasing, with the incidence reported as 50.1/100 000 population in 2019. (*2*) Data from *e-Wabak*, the communicable disease monitoring system run by the Ministry of Health in Malaysia, reported 46 outbreaks of foodborne disease in Seremban district, Negeri Sembilan state, between 2017 and 2021, with 18 (39.1%) involving educational institutions. Eleven (61.1%) of these outbreaks occurred at secondary schools, followed by 4 (22.2%) at primary schools and 3 (16.7%) at colleges. (*3*)

Food poisoning associated with *Bacillus cereus* causes diarrhoea or vomiting, (*4*) which is generally mild and self-limiting. Emetic syndrome develops upon ingesting the preformed toxin in contaminated foods such as rice or rice products. (*4*, *5*) Diarrhoeal syndrome, commonly associated with contaminated fish, vegetables or meat, is caused by heat-labile toxins, which have a longer incubation period. (*5*, *6*) The incubation period for the emetic syndrome is 0.5 to 6 hours, and for diarrhoeal syndrome it ranges from 8 to 16 hours. (*7*) *B. cereus* can withstand unfavourable conditions (*8*) and survives cooking processes, forming spores that can multiply and produce toxins in cooked rice. (*9*)

On 22 November 2021, the Seremban District Health Office was notified by a local hospital about cases of food poisoning occurring among boarding school students. The outbreak was confirmed by the rapid assessment team, consisting of doctors and environmental health assistant officers. The aim of this report is to describe the epidemiology of the outbreak, the steps taken to determine the causative pathogen and the contaminated foods and risk factors, and highlight recommendations to prevent future outbreaks.

## Methods

### Outbreak location

The outbreak occurred in a secondary-level boarding school attended by 597 female students in Seremban district, Negeri Sembilan state, an urban area in Malaysia.

### Epidemiological investigation

A case was defined as any person who developed abdominal pain, vomiting or diarrhoea after consuming any food or drinks at the boarding school canteen from 19 November 2021 at 06:30 to 21 November 2021 at 22:00. Active case-finding was conducted through face-to-face interviews, guided by a standardized Ministry of Health food poisoning questionnaire. Data were gathered about the sociodemographic characteristics of the cases, their symptoms, time of symptom onset and food consumption history. Records from government and private health-care facilities were reviewed to identify similar cases that may have been linked to the outbreak of foodborne illness through formal requests made to the relevant authorities.

An unmatched case–control study was conducted due to the challenges of recruiting participants during the coronavirus disease (COVID-19) pandemic and of conducting a field study. Individuals were eligible for inclusion if they had been exposed to the food prepared and served by the boarding school canteen during 19 to 21 November 2021. Participants were recruited using convenience sampling. The control group was defined as those who consumed the same food but did not experience any symptoms.

The attack rate of all students was determined, and the odds ratio (OR) was calculated for all reported food exposures. *P*-values were calculated using Fisher’s exact test, with statistical significance defined as *P* < 0.05. The data were verified by cross-checking with health-care facility and school records. Then the data were transformed into a line list using Microsoft Excel (2021). All data were collected in hardcopy form and kept confidential in locked storage by the Seremban District Health Office. Data were analysed using the IBM Statistical Package for the Social Sciences (SPSS) version 26 (Chicago, IL, USA).

### Laboratory investigation

Samples were collected for testing on 22 November 2021. Proxy food samples, hand swabs from food handlers and swabs from kitchen utensils were tested by culture and sensitivity for *B. cereus*, *Escherichia coli*, coliform bacteria, *Salmonella* species and *Staphylococcus aureus*. Rectal swabs from patients and food handlers were tested for enteric pathogens by culture and for sensitivity. Test results were obtained from the Public Health Laboratory Information System.

### Environmental investigation

An environmental investigation was conducted to assess the physical environment by examining factors such as the water source, food storage areas, sanitation practices, ventilation systems and other possible sources of contamination. The food preparation process was assessed using Hazard Analysis Critical Control Point standards. Food handlers were questioned about their typhoid vaccination status and their attendance at food handling courses, as guided by the Ministry of Health’s risk-based food premises inspection form, amendment 2018.

## Results

### Epidemiological investigation

A total of 152 cases were identified among the 597 students who lived at the boarding school, giving an attack rate of 25.5%. For the case–control study, there were 152 cases and 200 controls, for a ratio of 1:1.3. All cases were female and aged 13–17 years. They presented with abdominal pain (100%), nausea (97.4%, 148), vomiting (78.3%, 119) or diarrhoea (61.8%, 94), or a combination of these. All cases were treated as outpatients and none died.

**Fig. 1:**
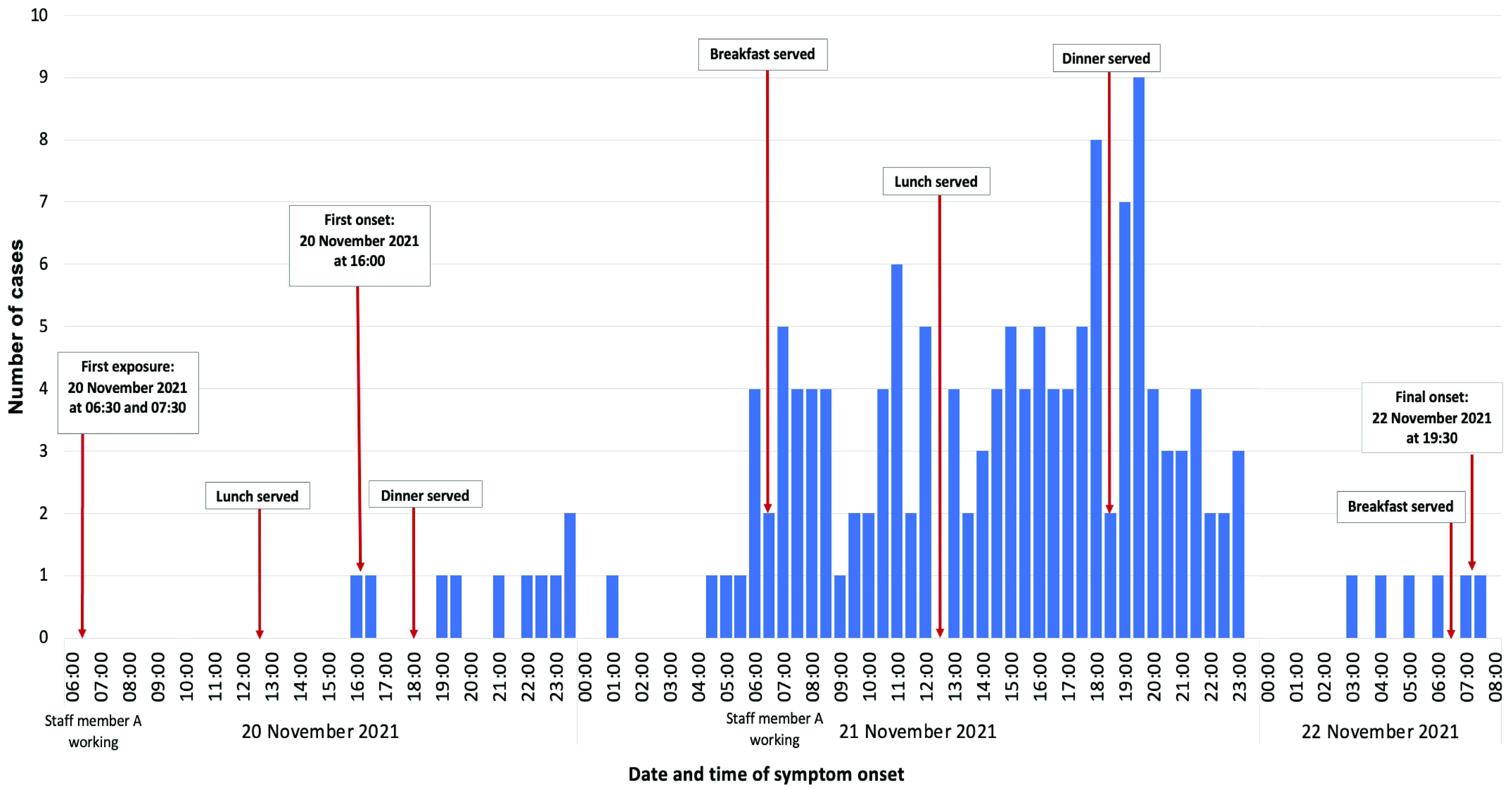
Epidemic curve of an outbreak of foodborne illness in a boarding school canteen, by date and time of symptom onset, Seremban district, Negeri Sembilan state, Malaysia, 20–22 November 2021

The epidemic curve suggests an outbreak with a continual source, as indicated by the multiple peaks (**Fig. 1**), with a suggested first exposure at breakfast on 20 November 2021. The epidemic curve also suggests that there was ongoing exposure because the onset of symptoms for the first case was on 20 November 2021 at 16:00, and the onset for the last case was on 22 November 2021 at 07:30.

[**insert** Figure 1]

The food served by the canteen operator during 20 and 21 November 2021 with the highest ORs included beef rendang and rice cubes, served during breakfast on 20 November (OR: 20.54, 95% CI: 4.89–86.30 for the beef rendang; OR: 19.62, 95% CI: 2.62–147.01 for the rice cubes); white rice served during lunch (OR: 9.64, 95% CI: 4.32–76.55); white rice and chicken curry served during dinner on the same day (OR: 19.62, 95% CI: 2.62–147.01); and vermicelli and spicy soy sauce served during breakfast on 21 November (OR: 17.02, 95% CI: 4.03–71.86) (**Table 1**). Leftover food was not consumed by students or staff.

**Table 1:** Case–control analysis of an outbreak of foodborne illness in a boarding school canteen, Seremban district, Negeri Sembilan state, Malaysia, 20–22 November 2021^a^

Type of food	Cases (*n* = 152)			Controls (*n* = 200)			Odds ratio (95% CI)	*P*
	Exposed	Not exposed	Total	Exposed	Not exposed	Total		
**20 November 2021 – Breakfast, 06:30 to 07:30**								
**Rice cubes^b^**	**150**	**2**	**152**	**161**	**39**	**200**	**18.17 (4.31–76.55)**	** < 0.001**
**Beef rendang^c^**	**150**	**2**	**152**	**157**	**43**	**200**	**20.54 (4.89–86.30)**	** < 0.001**
Peanut gravy	148	4	152	140	60	200	15.86 (5.61–44.78)	< 0.05
Malted chocolate drink	147	5	152	129	71	200	16.18 (6.34–41.31)	< 0.05
**20 November 2021 – Lunch, 12:30 to 13:30**								
**White rice**	**151**	**1**	**152**	**188**	**12**	**200**	**9.64 (1.24–74.96)**	** < 0.05**
**Fried spicy stuffed** **torpedo scad**	**151**	**1**	**152**	**187**	**13**	**200**	**10.50 (1.36–81.16)**	** < 0.05**
Turmeric fried prawns	150	2	152	181	19	200	7.87 (1.80–34.35)	< 0.05
Cabbage cooked in coconut broth	150	2	152	179	21	200	8.80 (2.03–38.14)	< 0.05
**20 November 2021 – Dinner, 18:00 to 19:00**								
**White rice**	**151**	**1**	**152**	**177**	**23**	**200**	**19.62 (2.62–147.01)**	** < 0.05**
**Chicken curry**	**151**	**1**	**152**	**177**	**23**	**200**	**19.62 (2.62–147.01)**	** < 0.05**
Stir fry vegetables	149	2	152	170	30	200	13.15 (3.09–55.95)	< 0.05
Red apple	146	6	152	170	30	200	4.29 (1.74–10.60)	< 0.05
Plain water	150	2	152	170	30	200	13.24 (3.11–56.32)	< 0.05
**21 November 2021 – Breakfast, 06:30 to 07:30**								
**Vermicelli**	**150**	**2**	**152**	**163**	**37**	**200**	**17.02 (4.03–71.86)**	** < 0.05**
**Spicy soy sauce**	**150**	**2**	**152**	**163**	**37**	**200**	**17.02 (4.03–71.86)**	** < 0.05**
Malted drinks	147	5	152	150	50	200	9.8 (3.80–25.27)	< 0.05

CI: confidence interval.

^a^ Foods in bold are suspected to have been associated with the outbreak.

^b^ Rice cubes are compacted rice that is boiled and subsequently cut into small cubes.

^c^ Beef rendang is a dry curry that is prepared using coconut milk and spices.

### Environmental investigation

The assessment of food processing showed a temperature monitoring violation, as there was no temperature monitoring when raw food was received or during its storage in the chiller and freezer. The beef was defrosted for less than 1 hour. The beef was boiled in 40-kg quantities in a cauldron without temperature monitoring for 20 minutes, which may have contributed to uneven and insufficient heating. The rice was washed before cooking and was cooked in a large pot, with approximately 5 kg rice per pot. It was cooked fresh for each meal and was not served again.

During inspection of the premises, flies were noted in the kitchen due to non-functioning fly traps. Hand-washing stations were not equipped with soap. Otherwise, the overall cleanliness of the premises was rated at 96.6%. All 14 food handlers were asymptomatic and had received typhoid vaccinations and attended food safety training.

### Laboratory investigation

The samples collected included five proxy foods (stir fry macaroni, white bread, fried chicken, soto vermicelli and chocolate cake), one sample of boiled water, 24 rectal swabs from 17 cases and seven food handlers, seven swabs from food preparers’ hands, seven environmental samples and 15 water samples from drinking-water dispensers at the school. The laboratory investigation yielded *B. cereus* (< 4000 colony forming units [CFU]/g) from the hand swab of one food preparer (staff member A) who worked the morning shifts on 20 and 21 November. Samples from two chopping boards also yielded *B. cereus* and *E. coli* (**Table 2**). The proxy food samples and the rice-scooping spoon yielded coliforms (**Table 2**).

**Table 2:** Laboratory results from an outbreak of foodborne illness in a boarding school canteen, Seremban district, Negeri Sembilan state, Malaysia, from samples collected on 22 November 2021

Type of specimen	Test and method	No. of samples	Test result		Organism identified
			Positive	Negative	
**Samples from cases and food handlers**					
Hand swabs (food handler)	Culture and sensitivity for enteric pathogens	7	1	6	*Bacillus cereus* (< 4 000 CFU/g) from staff member A
Rectal swabs (food handlers and cases)		24	0	24	–
**Samples from food and water**					
Stir fry macaroni	*B. cereus* (ISO 7 932:2004 [E])^a^ *Escherichia coli* (AOAC 991.14) Coliform (AOAC 991.14) *Salmonella species* (ISO 6 579–1:2017 [E]) *Staphylococcus aureus* (AOAC 2003.07–2006)	1	1	0	Coliform (1 300 CFU/g)
White bread		1	1	0	Coliform (30 000 CFU/g)
Fried chicken		1	0	1	–
Soto vermicelli^b^		1	0	1	–
Chocolate cake		1	0	1	–
Water (from drinking-water dispenser)	Coagulase-positive *Staphylococci* (ISO 6 888–1:1999/ Amd.1.2003 [E])	1	0	1	–
Drinking-water dispenser		1	0	1	–
Filtered water dispenser		1	0	1	–
**Samples from kitchen utensils**					
Cutting board (sample 1)	*B. cereus* (ISO 7 932:2004 [E]) *E. coli* (AOAC 991.14) Coliform (AOAC 991.14) *S. aureus* (AOAC 2003.07–2006)				*B. cereus* (< 4 000 CFU/g) *E. coli* (700 CFU/g) Coliform (350 000 CFU/g)
Cutting board (sample 2)		1	1	0	*B. cereus* (< 4 000 CFU/g) *E. coli* (100 CFU/g) Coliform (34 000 CFU/g)
Rice spoon		1	1	0	Coliform (6 500 CFU/g)
Ladle		1	0	1	–
Tip of water bottle		1	0	1	–
Pan surface		1	0	1	–

AOAC: Association of Official Analytical Collaboration International; CFU: colony forming units; ISO: International Organization for Standardization; –: indicates no findings.

^a^ The letter E after an ISO standard indicates that it was published in English.

^b^ Soto vermicelli is a plain soup eaten with vermicelli noodles.

### Outbreak control measures

Following the outbreak, all activities at the canteen were temporarily suspended in accordance with Malaysia’s Infectious Disease Control Act 1988. This allowed the kitchen operator to take necessary measures to ensure compliance with food and hygiene regulations before resuming operations. Food handlers were reminded to monitor temperatures closely, while the district health educator delivered talks to the food handlers and students as well as distributed informational pamphlets to students and teachers. To prevent future outbreaks, it is important to emphasize how to handle food safely and to educate food handlers, school administrators and students about food safety as well. A subsequent inspection was done to ensure that all necessary actions had been taken promptly.

## Discussion

Investigating outbreaks of foodborne illnesses enables possible sources of infection to be identified. This outbreak was most likely caused by *B. cereus* cross-contamination and poor temperature monitoring. In this outbreak, there were cases with emetic and diarrhoeal symptoms, suggesting a mixed source of *B. cereus*. (*5*)

The longer incubation periods observed and the continual common source pattern evidenced by the epidemiological curve, characterized by a gradual rise in the number of cases and numerous peaks over time, can be explained by multiple contamination incidents occurring over 2 days. The food could have been continually contaminated by staff member A, kitchen equipment or contaminated food. The self-service buffet style of food service during which students have their meals in groups in the dining hall may also have contributed to this type of epidemiological curve and could have facilitated possible contamination by students.

The ORs associated with the food should be interpreted with caution due to the wide confidence intervals, which make it difficult to determine which foods were most strongly associated with illness. However, not monitoring the temperature of the raw meat could also allow B. cereus to survive the cooking process and continue multiplying, (*10*) which could explain the high OR for beef rendang. Multiple factors commonly contribute to outbreaks of foodborne illness, especially those related to improper storage (i.e. refrigeration) and failure to maintain proper temperatures. (*11*) Other sauce-based dishes, such as chicken curry and spicy soy sauce, also had a high OR since they were served with rice. Rice was often served for breakfast, lunch and dinner on the dates around the outbreak occurrence, so it is possible that rice was also responsible for this outbreak.

Despite swabbing workers’ hands and kitchen equipment, only low numbers of CFUs were found; however, the positivity still indicates contamination since low numbers of *B. cereus* have caused outbreaks. (*12*) To improve the efficacy of hand-washing and reduce the number of *B. cereus* spores, soap should be used and hands should be washed for at least 20 seconds. (*13*, *14*) Drying hands with disposable towels instead of cloth towels can also reduce the risk of cross-contamination. (*15*) The presence of *E. coli* and coliforms suggests poor hygiene practices.

The study has limitations that make it difficult to definitively confirm that *B. cereus* caused the outbreak. None of the food items from the days on which contamination likely occurred were available for testing; instead, proxy food samples were used. When tested, these proxy samples were negative for *B. cereus* toxins. In terms of the statistical analyses, multivariable analysis was not performed, and the assessment of potentially contaminated food was conducted solely using univariable analysis. Ideally, a cohort study should have been conducted, but a case–control design was adopted due to the limited number of staff and stringent standard operating procedures implemented during the COVID-19 pandemic. Recall bias may exist due to the retrospective nature of participants providing a history of their food intake, and potential confounders were not adjusted for statistically. Due to the limitations identified in this study, the definitive causative agent of the foodborne outbreak is not confirmed.

In conclusion, this outbreak investigation highlights the need for proper food handling, temperature monitoring and hygiene practices. *B. cereus* most likely caused the outbreak through cross-contamination, while the presence of *E. coli* indicated poor hygiene standards. It is necessary to ensure there are strict enforcement of and more effective control measures, continual training and frequent spot inspections to identify and address any violations in food handling and hygiene practices.
